# Tasurgratinib (E7090) for cholangiocarcinoma with fibroblast growth factor receptor 2 fusions/rearrangements: a multicenter, open-label, Phase 2 study

**DOI:** 10.1093/jjco/hyaf119

**Published:** 2025-08-07

**Authors:** Lin Shen, Huaxin Duan, Takamichi Kuwahara, Taroh Satoh, Xuelei Ma, Sheng Yan, Haitao Zhao, Masafumi Ikeda, Tongjian Cui, Takashi Sasaki, Zhiqiang Meng, Yousuke Nakai, Makoto Ueno, Yoshito Komatsu, Hiroaki Nagano, Chigusa Morizane, Setsuo Funasaka, Hiroki Ikezawa, Takuya Nakada, Junji Furuse

**Affiliations:** State Key Laboratory of Holistic Integrative Management of Gastrointestinal Cancers, Beijing Key Laboratory of Cell & Gene Therapy for Solid Tumor, Department of Gastrointestinal Oncology, Peking University Cancer Hospital & Institute, 52 Fucheng Road, Haidian District, Beijing, 100142, China; Hunan Provincial People’s Hospital (The First Affiliated Hospital of Hunan Normal University), Department of Oncology, Hunan Hepatobiliary and Pancreatic Cancer Clinical Medical Research Center, Key Laboratory of Study and Discovery of Small Targeted Molecules of Hunan Province, 61 West Jiefang Road, Furong District, Changsha, Hunan, 410006, China; Aichi Cancer Center Hospital, Department of Gastroenterology, 1‐1 Kanokoden, Chikusa‐ku, Nagoya, Aichi, 464-8681, Japan; Center for Cancer Genomics and Precision Medicine, The University of Osaka Hospital, 2-2 Yamadaoka, Suita, Osaka, 565-0871, Japan; West China Hospital, Sichuan University, Department of Biotherapy, 37 Guoxue Lane, Wuhou District, Chengdu, Sichuan, 610041, China; The Second Affiliated Hospital, Zhejiang University, Department of Hepatobiliary and Pancreatic Surgery, 88 Jiefang Road, Shangcheng District, Hangzhou, Zhejiang, 310009, China; Peking Union Medical College Hospital, Department of Liver Surgery, 1 Shuaifuyuan Wangfujing, Dongcheng District, Beijing, 100006, China; National Cancer Center Hospital East, Department of Hepatobiliary and Pancreatic Oncology, 6-5-1 Kashiwanoha, Kashiwa, Chiba, 277-8577, Japan; Fujian Provincial Hospital, Department of Oncology, 134 Dongjie Street, Gulou District, Fuzhou, Fujian, 350001, China; Cancer Institute Hospital of JFCR, Department of Hepato-Biliary-Pancreatic Medicine, 3-8-31 Ariake, Koto-ku, Tokyo, 135-8550, Japan; Fudan University Shanghai Cancer Center, Department of Integrative Oncology, 270 Dongan Road, Xuhui District, Shanghai, 200032, China; Tokyo Women's Medical University, Department of Internal Medicine, Institute of Gastroenterology, 8-1 Kawada-cho, Shinjuku-ku, Tokyo, 162-8666, Japan; Kanagawa Cancer Center, Department of Gastroenterology, 2-3-2 Nakao, Asahi-ku, Yokohama, Kanagawa, 241-8515, Japan; Hokkaido University Hospital Cancer Center, Department of Cancer Chemotherapy, Kita 14, Nishi 5, Kita-ku, Sapporo, Hokkaido, 060-8648, Japan; Yamaguchi University Graduate School of Medicine, Department of Gastroenterological, Breast and Endocrine Surgery, 1-1-1 Minami-Kogushi, Ube, Yamaguchi, 755-8505, Japan; National Cancer Center Hospital, Department of Hepatobiliary and Pancreatic Oncology, 5-1-1 Tsukiji, Chuo-ku, Tokyo, 104-0045, Japan; Eisai Co., Ltd., Japan and Asia Clinical Development Department, 4-6-10 Koishikawa, Bunkyu-Ku, Tokyo, 112-8088, Japan; Eisai Co., Ltd., Japan and Asia Clinical Data Science Department, 4-6-10 Koishikawa, Bunkyu-Ku, Tokyo, 112-8088, Japan; Eisai Co., Ltd., Japan and Asia Clinical Development Department, 4-6-10 Koishikawa, Bunkyu-Ku, Tokyo, 112-8088, Japan; Kanagawa Cancer Center, Department of Gastroenterology, 2-3-2 Nakao, Asahi-ku, Yokohama, Kanagawa, 241-8515, Japan

**Keywords:** cholangiocarcinoma, gene fusion, gene rearrangement, in situ hybridization, fluorescence, receptor, fibroblast growth factor, type 2

## Abstract

**Background:**

This Phase 2 study (NCT04238715) evaluated the efficacy/safety of tasurgratinib 140 mg daily in patients with cholangiocarcinoma (CCA) and fibroblast growth factor receptor (*FGFR*) *2* fusions/rearrangements.

**Methods:**

Eligible Japanese and Chinese patients who had surgically unresectable, advanced, or metastatic CCA and had received ≥1 prior gemcitabine-based combination chemotherapy regimen were included and treated with oral tasurgratinib 140 mg daily. The primary endpoint was objective response rate (ORR); the study was considered successful if the lower limit of the ORRs 90% CI was >15%. Secondary endpoints included duration of response and safety. *FGFR2* fusions/rearrangements were confirmed by fluorescence in situ hybridization performed in central laboratories. Tumor responses were measured every 8 weeks by Response Evaluation Criteria in Solid Tumors version 1.1 per independent imaging review.

**Results:**

Sixty-three patients were treated; 23 (37%) had received 1 prior regimen, all others had received ≥2. By the data cutoff date (15 March 2023), the ORR was 30.2% (two-sided 90% CI: 20.7–41.0). The median duration of response for responders was 5.6 months (95% CI: 3.7–9.3; range: 1.0+ to 14.8+). Sixty-one patients (97%) had ≥1 treatment-related treatment-emergent adverse event; 18 patients (29%) had ≥1 grade ≥3 treatment-related treatment-emergent adverse events. Four patients (6%) had a fatal adverse event, none were considered treatment-related. Tasurgratinib had promising antitumor activity in patients with CCA harboring *FGFR2* fusions or rearrangements after ≥1 prior gemcitabine-based chemotherapy regimen.

**Conclusions:**

The primary endpoint (ORR) met the study’s predefined success criteria. Tasurgratinib had a manageable safety profile consistent with previous reports and the known pharmacological profile of FGFR inhibitors.

## Teaser

This Phase 2 study found that tasurgratinib 140 mg daily had promising antitumor activity, safety, and tolerability in patients with cholangiocarcinoma harboring fibroblast growth factor receptor 2 fusions/rearrangements after ≥1 prior gemcitabine-based chemotherapy regimen.

## Introduction

Cholangiocarcinoma (CCA) has been estimated to comprise ~12% of intrahepatic cancers [1]; further, among all biliary tract cancers (including intrahepatic and extrahepatic CCAs, gallbladder cancer, and cancer of the Ampulla of Vater), ~39% of cases are intrahepatic CCA [2]. Outcomes for patients with CCA tend to be poor, owing to difficulties in diagnosis—as the symptoms of CCA are relatively nonspecific, patients tend to present with late stages of the disease [3].

Surgery is the primary treatment option for CCA [4]; treatment options for patients with unresectable or metastatic CCA are thus limited—gemcitabine plus cisplatin with durvalumab is currently recommended as first-line treatment for unresectable or metastatic CCA. Although FOLFOX and a regimen of 5-fluorouracil, leucovorin, and irinotecan have been evaluated as second-line treatment of advanced biliary tract cancer, second-line care has not yet been standardized [4].

Treatment decisions for CCA are also guided by the presence of gene alterations, such as in fibroblast growth factor receptor (*FGFR*) 2, isocitrate dehydrogenase (*IDH*) 1 and 2, *KRAS*, and *BRAF* [5–7]; in particular, *FGFR2* fusion detected in patients with CCA generally appear to be mutually exclusive with *KRAS* and *BRAF* mutations [8], similar to gastric cancer [9]. The fibroblast growth factor family of genes encodes for a variety of signaling proteins that function through tyrosine kinase receptors FGFR 1–4; they are ubiquitous and control a variety of biological functions including metabolism, cell differentiation/proliferation/apoptosis, and regulation of tissue fibrosis [10]. In particular, *FGFR2* fusions or rearrangements occur in ~5%–15% of intrahepatic CCA cases and ~4% of perihilar CCA cases [10–15]. Thus, the inhibition of FGFR2 is a promising approach for developing therapies for CCA in the presence of *FGFR2* fusions or rearrangements.

For patients who fail to respond to first-line cisplatin/gemcitabine/durvalumab therapy and who show presence of *FGFR2* fusions or rearrangements, FGFR inhibitors (including pemigatinib and futibatinib) are recommended [16]. Next-generation sequencing (NGS) is typically used as a companion diagnostic tool to determine *FGFR2* fusions or rearrangements [17, 18]. NGS has some notable drawbacks: generally, NGS requires at least 10 formalin-fixed paraffin-embedded (FFPE) slides [19, 20], which may cause complications if patients cannot provide enough tumor sample for evaluation. Further, the complexity of NGS testing requires samples be delivered to a testing facility, which may induce further delays until test results are available [21].

Tasurgratinib (also known as E7090) is an orally available selective inhibitor of FGFR 1–3 and was recently approved in Japan for biliary tract cancers with FGFR2 fusions or rearrangements [22, 23]. In contrast to other FGFR inhibitors, tasurgratinib is paired with fluorescence in situ hybridization (FISH) testing to confirm *FGFR2* fusions or rearrangements. The recommended dose of tasurgratinib was 140 mg per day per the dose-escalation part of a first-in-human Phase 1 study (NCT02275910) [24, 25]. In this pivotal Phase 2 study (NCT04238715), this regimen was evaluated in patients with CCA and *FGFR2* fusions or rearrangements.

## Methods

### Study design and patients

This open-label study enrolled Japanese and Chinese patients with surgically unresectable advanced or metastatic CCA to receive oral tasurgratinib 140 mg daily in 28-day cycles. This study also utilized an epidemiology study in Japan (jRCT1080225086) [26] to identify patients with *FGFR2* fusion-positive CCA and accelerate enrollment of this rare subpopulation. Patients needed to have CCA with *FGFR2* fusions or rearrangements and needed to have been treated with ≥1 prior chemotherapy regimen including a gemcitabine-based combination. Additionally, patients could not have received FGFR2 inhibitors. Tasurgratinib was provided by the sponsor as 35 mg tablets.

This study was conducted in accordance with standard operating procedures of the sponsor, which were based on the Principles of the World Medical Association Declaration of Helsinki, International Council for Harmonisation of Technical Requirements for Pharmaceuticals for Human Use (ICH) Good Clinical Practice, and all applicable regulations in Japan and China. Ethical approval and written informed consent were granted and approved by the applicable local institutional review board. Signed informed consent forms were obtained from each patient prior to enrollment.

### Endpoints

The primary endpoint was objective response rate (ORR), assessed per Response Evaluation Criteria in Solid Tumors (RECIST) version 1.1 by independent imaging review. Secondary endpoints included duration of response (DOR), time-to-response (TTR), disease control rate (DCR), clinical benefit rate (CBR), and progression-free survival (PFS) per RECIST version 1.1 by independent imaging review, overall survival (OS), and safety.

### Assessments

Tumor responses were measured every 8 weeks. Assessments of complete response (CR) and partial response (PR) required subsequent confirmation at least 28 days later. ORR was calculated as the proportion of patients who had a CR or PR. DCR was calculated as the proportion of patients who had a CR, PR, or stable disease (SD) for ≥7 weeks from the first dose of study drug received; and CBR was calculated as the proportion of patients who had a CR, PR, or SD for ≥23 weeks from the first dose of study drug.

Treatment-emergent adverse events (TEAEs) were coded using the Medical Dictionary for Regulatory Activities version 26.0; severity was measured per Clinical Trial Criteria for Adverse Events version 4.03. Hyperphosphatemia was assessed per protocol by phosphate level for dose modification purposes: for phosphate levels 5.5–7.0 mg/dl, treatment for hyperphosphatemia was to be initiated per the investigator’s discretion. For phosphate levels 7.1–9.0 mg/dl for 2 weeks, or levels ≥9.1 mg/dl despite optimal treatment, tasurgratinib was to be interrupted until phosphate levels returned to ≤7.0 mg/dl and dose reduced by 1 dose level.

Genetic testing for *FGFR2* alteration was performed in central laboratories using a break apart FISH probe kit (5′ flank and 3′ flank were labeled green and orange, respectively) with 2–4 FFPE slides; results were to be made available within 2 weeks.

### Statistical analysis

A sample size of 60–64 patients was planned; the study was considered successful if the lower limit of the ORR 90% CI was >15% in the planned population, corresponding to at least 15 confirmed responses. Confidence intervals (CIs) for tumor responses were calculated using the Clopper–Pearson method. PFS, DOR, TTR, and OS were summarized and plotted by the Kaplan–Meier method, and 95% CIs were presented.

## Results

### Patients

Sixty-three patients (Japanese: *n* = 28; Chinese: *n* = 35) were treated (median age: 55 years); 23 patients (36.5%) had received 1 prior regimen; all others had received ≥2 regimens (Table 1). Of the treated patients, 33 (52.4%) were female and 30 (47.6%) were male; 17 of the 33 female patients (51.5%) and 6 of the 30 male patients (20.0%) had received 1 prior regimen. By the data cutoff date (15 March 2023), 55 patients had discontinued treatment, primarily due to either disease progression (*n* = 48), adverse events (*n* = 4), patient choice (*n* = 2), or loss of clinical benefit (*n* = 1). The median follow-up time was 13.1 months (range 1.1+ to 34.3).

**Table 1:** Patient baseline characteristics.

**Category**	**Japanese (*n* = 28)**	**Chinese (*n* = 35)**	**Overall (*N* = 63)**
**Histology**, *n* (%)			
Intrahepatic	26 (92.9)	35 (100)	61 (96.8)
Perihilar	2 (7.1)	0	2 (3.2)
**Median age**, years (range)	66.5 (37–80)	53.0 (33–71)	55.0 (33–80)
**Age, years**, patient *n* (%)			
<65 years	11 (39.3)	32 (91.4)	43 (68.3)
≥65 years	17 (60.7)	3 (8.6)	20 (31.7)
**Sex**, *n* (%)			
Male	17 (60.7)	13 (37.1)	30 (47.6)
Female	11 (39.3)	22 (62.9)	33 (52.4)
**ECOG PS**, *n* (%)			
0	17 (60.7)	14 (40.0)	31 (49.2)
1	11 (39.3)	21 (60.0)	32 (50.8)
**HBsAg**, *n* (%)			
Positive	1 (3.6)	10 (28.6)	11 (17.5)
Negative	27 (96.4)	25 (71.4)	52 (82.5)
**HCVAb**, *n* (%)			
Positive	2 (7.1)	0	2 (3.2)
Negative	26 (92.9)	35 (100.0)	61 (96.8)
**Patients per no. of prior systemic anticancer medication regimens**, *n* (%)			
1	12 (42.9)	11 (31.4)	23 (36.5)
2	9 (32.1)	11 (31.4)	20 (31.7)
3	6 (21.4)	6 (17.1)	12 (19.0)
4	0	6 (17.1)	6 (9.5)
≥5	1 (3.6)	1 (2.9)	2 (3.2)
**Patients with a prior ICI,** *n* (%)			
Yes	1 (3.6)	19 (54.3)	20 (31.7)
No	27 (96.4)	16 (45.7)	43 (68.3)

ECOG PS, Eastern Cooperative Oncology Group performance status; HCVAb, hepatitis C antibody; HBsAg, hepatitis B surface antigen; ICI, immune checkpoint inhibitor.

### Efficacy

Nineteen patients (30.2%) had a PR and 31 patients (49.2%) had SD (Table 2 and Fig. S1); the ORR was 30.2% (two-sided 90% CI: 20.7–41.0; 95% CI 19.2–43.0) (Table 2), the DCR was 79.4% (95% CI: 67.3–88.5), and the CBR was 50.8% (95% CI: 37.9–63.6). The median TTR for responders was 1.87 months (IQR 1.77–2.10; range 1.6–14.7), and their median DOR was 5.6 months (95% CI 3.7–9.3; range 1.0+ to 14.8+).

**Table 2:** Summary of tumor response per RECIST version 1.1 by independent imaging review.

**Category**	**Japanese (*n* = 28)**	**Chinese (*n* = 35)**	**Overall (*N* = 63)**
**Best overall response**, *n* (%)			
CR	0	0	0
PR	7 (25.0)	12 (34.3)	19 (30.2)
SD ≥7 weeks after the first dose	13 (46.4)	18 (51.4)	31 (49.2)
SD ≥23 weeks after first dose	5 (17.9)	8 (22.9)	13 (20.6)
PD	8 (28.6)	4 (11.4)	12 (19.0)
Not evaluable	0	1 (2.9)	1 (1.6)
**Objective response rate**, *n* (%)	7 (25.0)	12 (34.3)	19 (30.2)
(90% CI)	(12.4–41.9)	(21.1–49.6)	(20.7–41.0)
(95% CI)	(10.7–44.9)	(19.1–52.2)	(19.2–43.0)
**Disease control rate**, *n* (%)	20 (71.4)	30 (85.7)	50 (79.4)
**Clinical benefit rate**, *n* (%)	12 (42.9)	20 (57.1)	32 (50.8)
**Median time to response**, months	1.87	1.87	1.87
(IQR)^a^	(1.77–3.71)	(1.79–2.02)	(1.77–2.10)
Range	1.6–14.7	1.6–7.4	1.6–14.7
**Median duration of response**, months			
(95% CI)	5.7 (3.7–9.3)	5.5 (3.5–10.2)	5.6 (3.7–9.3)
Range	3.7 to 14.8+	1.0+ to 11.1	1.0+ to 14.8+

CI, confidence interval; CR, complete response; IQR, interquartile range; PD, progressive disease; PR, partial response; RECIST, Response Evaluation Criteria in Solid Tumors; SD, stable disease.

^a^For responders.

Patients’ duration of treatment and tumor assessments by timepoint are shown in Fig. S2. Of the 23 patients who received only 1 prior systemic anticancer therapy regimen, 9 had an ORR (39.1%); ORRs in patients who received 2 prior regimens and in those who received ≥3 prior regimens were both 25.0%, corresponding to 5/20 responders each (Table 3). Of the 20 patients who received a prior immune checkpoint inhibitor, 4 had a partial response for an ORR of 20.0%. Patients with an Eastern Cooperative Oncology Group performance status (ECOG PS) of 0 had an ORR of 35.5% (11/31 patients), while those with an ECOG PS of 1 had an ORR of 25.0% (8/32); the ORR was generally consistent by age group.

**Table 3:** Subgroup analysis of objective response rate.

**Category**	**Included patients**, *n*	**Responders**, *n*^a^	**Objective response rate**, % (95% CI)^a^^,^^b^
**Overall**	63	19	30.2 (19.2–43.0)
**Age group (years)**			
<65	43	13	30.2 (17.2–46.1)
≥65	20	6	30.0 (11.9–54.3)
**Sex**			
Male	30	6	20.0 (7.7–38.6)
Female	33	13	39.4 (22.9–57.9)
**ECOG PS**			
0	31	11	35.5 (19.2–54.6)
1	32	8	25.0 (11.5–43.4)
**Number of prior systemic anticancer regimens**			
1	23	9	39.1 (19.7–61.5)
2	20	5	25.0 (8.7–49.1)
≥3	20	5	25.0 (8.7–49.1)
**Receipt of ICI therapies**			
Yes	20	4	20.0 (5.7–43.7)
No	43	15	34.9 (21.0–50.9)

CI, confidence interval; ECOG PS, Eastern Cooperative Oncology Group performance status; ICI, immune checkpoint inhibitor; RECIST, Response Evaluation Criteria in Solid Tumors.

^a^Per RECIST version 1.1 by independent imaging review.

^b^Proportion of responders among all patients per baseline characteristic.

**Figure 1:**
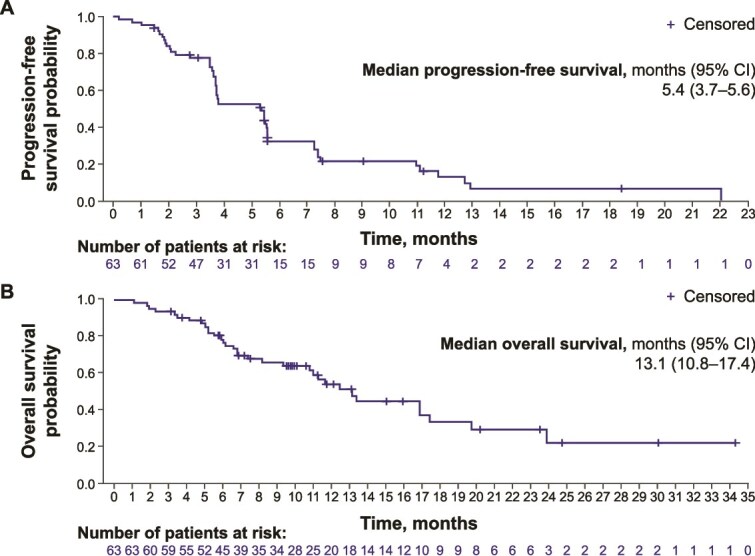
Median progression-free survival^a^ (A) and overall survival (B). ^a^Per RECIST version 1.1 by independent imaging review. CI, confidence interval; RECIST, Response Evaluation Criteria in Solid Tumors.

The median PFS was 5.4 months (95% CI: 3.7–5.6) (Fig. 1A), and the median OS was 13.1 months (95% CI: 10.8–17.4) (Fig. 1B). Median PFS and OS are summarized in Table S1.

### Safety

All patients (*N* = 63) had ≥1 TEAE (Table S2). Sixty-one patients (96.8%) had ≥1 treatment-related TEAE, most commonly hyperphosphatemia (*n* = 51; 81%); 18 patients (28.6%) had ≥1 grade ≥3 treatment-related TEAE, most commonly lipase increased (*n* = 4; 6.3%) (Table 4). Seventeen patients (27.0%) received phosphate binders to treat hyperphosphatemia.

**Table 4:** Treatment-related TEAEs occurring in ≥15% of all patients.

**Category**, *n* (%)	**Japanese (*n* = 28)**		**Chinese (*n* = 35)**		**Overall (*N* = 63)**	
	**Any grade**	**Grade ≥3**	**Any grade**	**Grade ≥3**	**Any grade**	**Grade ≥3**
**Patients with any treatment-related TEAEs**	26 (92.9)	7 (25.0)	35 (100)	11 (31.4)	61 (96.8)	18 (28.6)
Hyperphosphatemia	21 (75.0)	2 (7.1)	30 (85.7)	1 (2.9)	51 (81.0)	3 (4.8)
PPES	14 (50.0)	2 (7.1)	14 (40.0)	0	28 (44.4)	2 (3.2)
Diarrhea	10 (35.7)	0	10 (28.6)	0	20 (31.7)	0
Paronychia	11 (39.3)	0	3 (8.6)	0	14 (22.2)	0
Stomatitis	12 (42.9)	1 (3.6)	2 (5.7)	0	14 (22.2)	1 (1.6)
AST increased	1 (3.6)	0	11 (31.4)	0	12 (19.0)	0
Dry mouth	5 (17.9)	0	7 (20.0)	0	12 (19.0)	0
Nail discoloration	4 (14.3)	0	8 (22.9)	0	12 (19.0)	0
Onycholysis	7 (25.0)	0	5 (14.3)	0	12 (19.0)	0
ALT increased	1 (3.6)	0	10 (28.6)	1 (2.9)	11 (17.5)	1 (1.6)
Dysgeusia	8 (28.6)	0	2 (5.7)	0	10 (15.9)	0
Lipase increased	5 (17.9)	2 (7.1)	5 (14.3)	2 (5.7)	10 (15.9)	4 (6.3)

ALT, alanine aminotransferase; AST, aspartate aminotransferase; PPES, palmar-plantar erythrodysesthesia syndrome; TEAE, treatment-emergent adverse event.

Thirty-four patients (54.0%) had a dose reduction due to treatment-related TEAEs, most commonly (*n* ≥ 2): palmar-plantar erythrodysesthesia syndrome (*n* = 8; 12.7%), hyperphosphatemia (*n* = 4; 6.3%), paronychia (*n* = 4; 6.3%), corneal epithelium defect, diarrhea, keratitis, and retinal detachment (each *n* = 3; 4.8%), and vision blurred, corneal opacity, nail bed bleeding, onychalgia, onycholysis, and stomatitis (each *n* = 2; 3.2%). Eighteen patients (28.6%) had treatment interruption due to treatment-related TEAEs. Three patients (4.8%) withdrew from receiving tasurgratinib due to treatment-related TEAEs, which were corneal epithelium defect, hemoptysis, and stomatitis (*n* = 1 each).

Four patients (6.3%) had a fatal TEAE; however, none were related to treatment. These were recorded as myocardial ischemia, multiple organ dysfunction syndrome (each in one patient), and death (not otherwise specified, in two patients).

The most common treatment-related TEAEs (≥30%) by baseline characteristics (i.e. age < or ≥65 years; ECOG PS 0 or 1; and number of prior therapy regimens) are reported in Table S3. Notably, hyperphosphatemia was the most frequent treatment-related TEAE, irrespective of baseline subgroup analyzed.

## Discussion

Tasurgratinib had promising antitumor activity in patients with CCA harboring *FGFR2* fusions or rearrangements who received ≥1 prior gemcitabine-based chemotherapy regimen. The primary endpoint (ORR) was 30.2%; it met the study’s predefined success criteria. Tasurgratinib also retained antitumor activity in more heavily pretreated patients; the ORR of patients who received ≥3 systemic anticancer regimens was 25.0%. The ORR was lower in male patients than female patients, but this may be because male patients were more heavily pretreated than female patients; 20.0% of male patients (6/30) received only a single line of prior therapy compared with 51.5% of female patients (17/33).

Tasurgratinib had a manageable overall safety profile. Observed hepatic events (including increases in alanine aminotransferase and aspartate aminotransferase) may be attributed to the study disease indication, comorbid conditions, and disease progression. Nail toxicities including paronychia, nail discoloration, and nail bed bleeding were reported, similar to other FGFR inhibitors [27].

Acknowledging the limitations of cross-study comparisons, tasurgratinib demonstrated higher antitumor activity compared with historical data of chemotherapy as first- to third-line treatment [28–30]. Tasurgratinib had an ORR of 30.2%, which was comparable to the FGFR inhibitors pemigatinib (ORR: 35.5%), futibatinib (ORR: 42%), and infigratinib (ORR: 23.1%) [17, 18, 31] when accounting for previous therapies received. A greater proportion of patients in this trial (31.7%) had received three or more anticancer therapies than in these other trials (12%–23%) [17, 18]; notably, historical data and retrospective analyses indicate that the efficacy of chemotherapy declines in later lines of treatment [28–30]. The ORR of tasurgratinib in patients who received only one line of prior therapy was 39.1%, compared with 36.9% [17] for pemigatinib and 37.5% [18] for futibatinib. Tasurgratinib also showed a manageable safety profile consistent with previous reports [25] and with the known pharmacological profile of FGFR inhibitors [17, 18, 31]. Thus, the efficacy and safety of tasurgratinib appear to be comparable to pemigatinib and futibatinib. Conclusions made from this trial are limited by the trial’s single-arm design and the limited patient sample size.

FGFR inhibitors have generally been considered second-line treatments for CCA (with molecular assessment during or following first-line chemotherapy) [16]; however, Phase 3 studies of pemigatinib, futibatinib, and infigratinib as first-line therapies for patients harboring *FGFR2* fusions or rearrangements have been attempted [32–34]. Although these studies have had challenges with enrollment (occasionally leading to study termination), some preliminary antitumor activity was observed with infigratinib over gemcitabine/cisplatin [35]. FGFR inhibitors have the potential to shift the treatment paradigm for this disease, warranting earlier and more efficient molecular assessment of *FGFR2* status. This study assessed *FGFR2* fusions or rearrangements by FISH instead of NGS, in contrast to other studies of FGFR inhibitors [17, 18]. FISH testing in our trial required a lower number of FFPE slides [2–4] than typical NGS tests (10 [19, 20]); further, its availability to be conducted at a larger number of clinical sites may help decrease turnaround time: for NGS, the process of transporting tumor samples to be assessed by an expert panel causes additional delays until results are available to the clinician [21]. Thus, FISH may benefit patients who cannot afford delays to starting treatment or patients who have few viable tumor samples accessible for genetic testing.

*FGFR2* mutations are known to occur after treatment with FGFR inhibitors as a mechanism of acquired resistance in CCA; of particular note are *FGFR2, N549K*, and *N549H* mutations, which have been reported to occur after treatment with pemigatinib and futibatinib [36, 37]. In a preclinical model, tasurgratinib demonstrated growth-inhibitory activity in the presence of *FGFR2, N549K*, and *N549H* [38]. Tasurgratinib has also been reported to exhibit type V binding kinetics to FGFR (fast association and slow dissociation) [22], which is believed to contribute to its high affinity even against secondary *FGFR2* resistance mutations. Thus, preclinical evidence suggests that tasurgratinib may demonstrate clinical activity against CCA resistant to pemigatinib and futibatinib.

Pemigatinib [39] and futibatinib [40] are known to be metabolized by CYP3A, and thus concomitant use with CYP3A inducers or inhibitors should be avoided. Preclinical studies of tasurgratinib (Eisai Co., Ltd., data on file) have suggested that CYP4F12 is primarily involved in the conversion of tasurgratinib into its active metabolite M2. As no clinical inhibitors and inducers of CYP4F12 are currently known, this has the potential to simplify dose administration and therapeutic management.

In summary, tasurgratinib demonstrated clinical efficacy in pretreated CCA with *FGFR2* fusions or rearrangements comparable with other FGFR inhibitors and was tolerable. Considering the availability of FISH genetic testing, and potential therapeutic roles in the presence of *FGFR2* resistance mutations or the possibility of coadministration with CYP3A inhibitors/inducers, tasurgratinib has practical applications and may be a beneficial future addition to current treatment paradigms for CCA with *FGFR2* fusions or rearrangements.

## Supplementary Material

hyaf119_Supplementary_Figure_S1_hyaf119

hyaf119_Supplementary_Figure_S2_hyaf119

hyaf119_Supplementary_Table_S1_hyaf119

hyaf119_Supplementary_Table_S2_hyaf119

hyaf119_Supplementary_Table_S3_hyaf119

## Data Availability

These data will not be available for sharing at this time because these data are commercially confidential. However, Eisai Inc. will consider written requests to share the data on a case-by-case basis.

## Notes

**Trial registration:**  Clinicaltrials.gov  NCT04238715

**Previous presentation:** These results were presented in part at the American Society for Clinical Oncology Gastrointestinal Cancers Symposium held on 18–20 January 2024.
