# Supplementary material for: Tasurgratinib (E7090) for cholangiocarcinoma with fibroblast growth factor receptor 2 fusions/rearrangements: a multicenter, open-label, Phase 2 study
Source: Jpn J Clin Oncol. 2025 Aug 7;55(11):1229–36. doi: 10.1093/jjco/hyaf119 (PMC12598627; doi:10.1093/jjco/hyaf119)
Supplement: hyaf119_Supplementary_Figure_S2_hyaf119 [file hyaf119_supplementary_figure_s2_hyaf119.pdf]

Fig. S2. Patients' duration of treatment and tumor assessments per RECIST v1.1 by independent imaging review

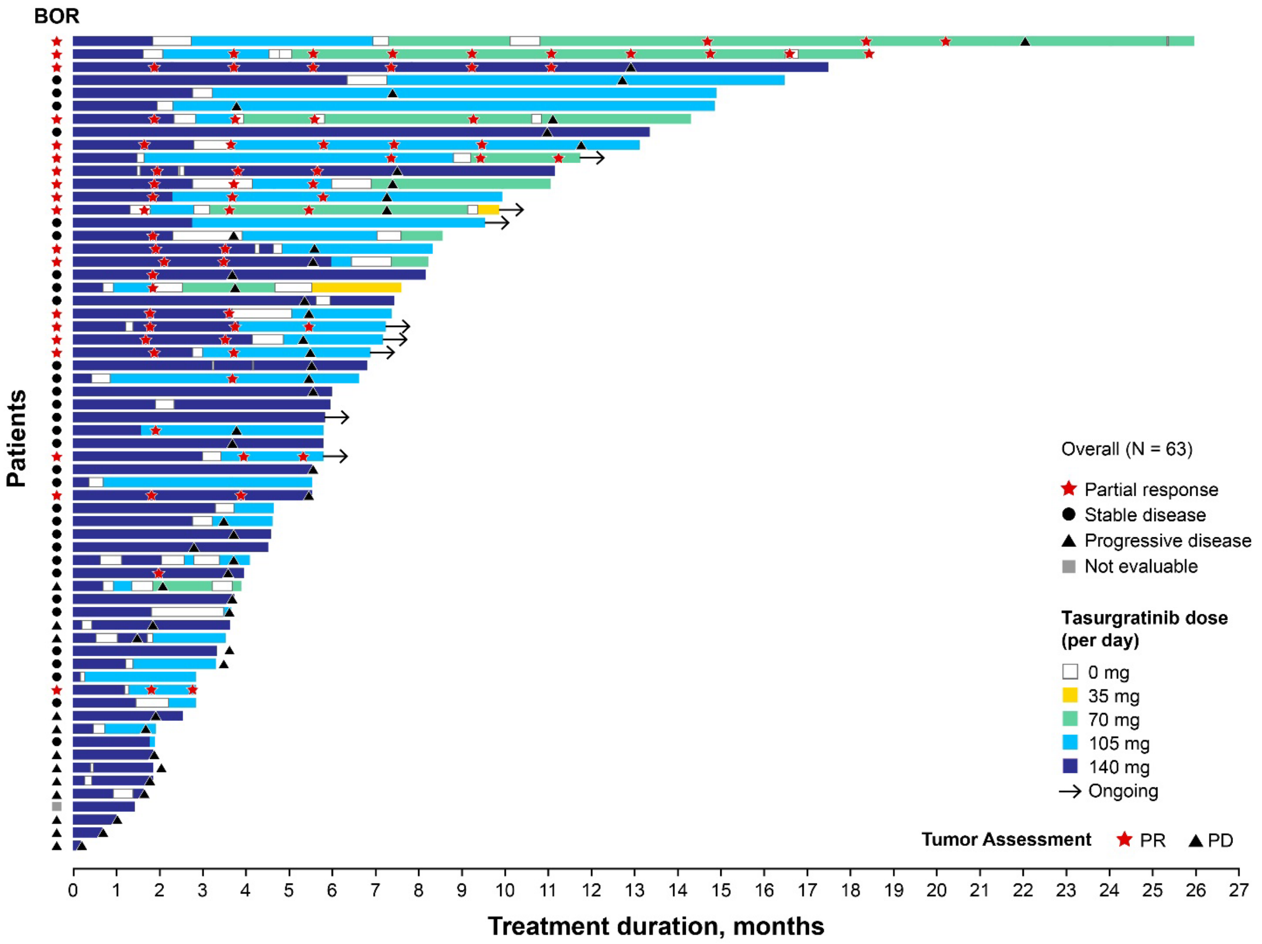

BOR, best overall response; PD, progressive disease; PR, partial response; RECIST v1.1, Response Evaluation Criteria in Solid Tumors version 1.1.
