# Supplementary material for: Tasurgratinib (E7090) for cholangiocarcinoma with fibroblast growth factor receptor 2 fusions/rearrangements: a multicenter, open-label, Phase 2 study
Source: Jpn J Clin Oncol. 2025 Aug 7;55(11):1229–36. doi: 10.1093/jjco/hyaf119 (PMC12598627; doi:10.1093/jjco/hyaf119)
Supplement: hyaf119_Supplementary_Table_S1_hyaf119 [file hyaf119_supplementary_table_s1_hyaf119.pdf]

Table S1. Progression-free survival and overall survival by country

Category	Japanese (n=28)	Chinese (n=35)	Overall (N=63)
**Median progression-free survival**,[a] months	3.6	5.5	5.4
95% CI	2.3–7.3	3.7–5.6	3.7–5.6
**Median overall survival**, months	10.8	16.9	13.1
95% CI	6.0–17.4	11.0–NE	10.8–17.4

[a]Per RECIST v1.1 by independent imaging review.

CI, confidence interval; NE, not estimable; RECIST v1.1, Response Evaluation Criteria In Solid Tumors version 1.1.
