# Supplementary material for: Tasurgratinib (E7090) for cholangiocarcinoma with fibroblast growth factor receptor 2 fusions/rearrangements: a multicenter, open-label, Phase 2 study
Source: Jpn J Clin Oncol. 2025 Aug 7;55(11):1229–36. doi: 10.1093/jjco/hyaf119 (PMC12598627; doi:10.1093/jjco/hyaf119)
Supplement: hyaf119_Supplementary_Table_S2_hyaf119 [file hyaf119_supplementary_table_s2_hyaf119.pdf]

Table S2. TEAEs occurring in ≥15% of all patients

**Category**, n (%)	**Japanese (n=28)**		**Chinese (n=35)**		**Overall (N=63)**	
	**Any grade**	**Grade ≥3**	**Any grade**	**Grade ≥3**	**Any grade**	**Grade ≥3**
**Patients with any TEAEs**	28 (100)	17 (60.7)	35 (100)	18 (51.4)	63 (100)	35 (55.6)
Hyperphosphatemia	21 (75.0)	2 (7.1)	30 (85.7)	1 (2.9)	51 (81.0)	3 (4.8)
PPES	14 (50.0)	2 (7.1)	14 (40.0)	0	28 (44.4)	2 (3.2)
Diarrhea	12 (42.9)	0	11 (31.4)	0	23 (36.5)	0
AST increased	6 (21.4)	1 (3.6)	14 (40.0)	1 (2.9)	20 (31.7)	2 (3.2)
ALT increased	4 (14.3)	1 (3.6)	14 (40.0)	1 (2.9)	18 (28.6)	2 (3.2)
Dry mouth	6 (21.4)	0	8 (22.9)	0	14 (22.2)	0
Stomatitis	14 (50.0)	1 (3.6)	2 (5.7)	0	16 (25.4)	1 (1.6)
Paronychia	11 (39.3)	0	3 (8.6)	0	14 (22.2)	0
Pyrexia	10 (35.7)	0	4 (11.4)	0	14 (22.2)	0
Keratitis	8 (28.6)	0	5 (14.3)	0	13 (20.6)	0
Blood ALP increased	3 (10.7)	1 (3.6)	10 (28.6)	1 (2.9)	13 (20.6)	2 (3.2)
Blood creatinine increased	5 (17.9)	0	8 (22.9)	0	13 (20.6)	0
Lipase increased	8 (28.6)	3 (10.7)	5 (14.3)	2 (5.7)	13 (20.6)	5 (7.9)
Onycholysis	7 (25.0)	0	5 (14.3)	0	12 (19.0)	0
Nail discoloration	4 (14.3)	0	8 (22.9)	0	12 (19.0)	0
Dysgeusia	9 (32.1)	0	2 (5.7)	0	11 (17.5)	0
Nail bed bleeding	7 (25.0)	0	3 (8.6)	0	10 (15.9)	0
Neutrophil count decreased	3 (10.7)	0	7 (20.0)	2 (5.7)	10 (15.9)	2 (3.2)
WBC count decreased	1 (3.6)	0	9 (25.7)	1 (2.9)	10 (15.9)	1 (1.6)

ALP, alkaline phosphatase; ALT, alanine aminotransferase; AST, aspartate aminotransferase; PPES, palmar-plantar erythrodysesthesia syndrome; TEAE, treatment-emergent adverse event; WBC, white blood cell.
