# Supplementary material for: Tasurgratinib (E7090) for cholangiocarcinoma with fibroblast growth factor receptor 2 fusions/rearrangements: a multicenter, open-label, Phase 2 study
Source: Jpn J Clin Oncol. 2025 Aug 7;55(11):1229–36. doi: 10.1093/jjco/hyaf119 (PMC12598627; doi:10.1093/jjco/hyaf119)
Supplement: hyaf119_Supplementary_Table_S3_hyaf119 [file hyaf119_supplementary_table_s3_hyaf119.pdf]

Table S3. Treatment-related TEAEs occurring in ≥30% of patients in any baseline characteristic subgroup

Patient Age	Age <65 years (n=43)		Age ≥65 years (n=20)	
	Any grade	Grade ≥3	Any grade	Grade ≥3
**Patients with any treatment-related TEAEs**, n (%)	42 (97.7)	11 (25.6)	19 (95.0)	7 (35.0)
Hyperphosphatemia	37 (86.0)	1 (2.3)	14 (70.0)	2 (10.0)
PPES	19 (44.2)	0	9 (45.0)	2 (10.0)
Diarrhea	14 (32.6)	0	6 (30.0)	0
Paronychia	8 (18.6)	0	6 (30.0)	0
Stomatitis	7 (16.3)	0	7 (35.0)	1 (5.0)

ECOG PS	ECOG PS 0 (n=31)		ECOG PS 1 (n=32)	
	Any grade	Grade ≥3	Any grade	Grade ≥3
**Patients with any treatment-related TEAEs**	31 (100.0)	7 (22.6)	30 (93.8)	11 (34.4)
Hyperphosphatemia	23 (74.2)	0	28 (87.5)	3 (9.4)
PPES	17 (54.8)	2 (6.5)	11 (34.4)	0
Diarrhea	11 (35.5)	0	9 (28.1)	0
Onycholysis	10 (32.3)	0	2 (6.3)	0
Stomatitis	10 (32.3)	1 (3.2)	4 (12.5)	0

Prior therapy regimens	1 Prior therapy (n=23)		2 Prior therapies (n=20)		≥3 Prior therapies (n=20)	
	Any grade	Grade ≥3	Any grade	Grade ≥3	Any grade	Grade ≥3
**Patients with any treatment-related TEAEs**	21 (91.3)	7 (30.4)	20 (100.0)	6 (30.0)	20 (100.0)	5 (25.0)
Hyperphosphatemia	18 (78.3)	0	14 (70.0)	1 (5.0)	19 (95.0)	2 (10.0)
PPES	9 (39.1)	1 (4.3)	10 (50.0)	1 (5.0)	9 (45.0)	0
Diarrhea	7 (30.4)	0	7 (35.0)	0	6 (30.0)	0
Stomatitis	7 (30.4)	1 (4.3)	3 (15.0)	0	4 (20.0)	0
Onycholysis	3 (13.0)	0	3 (15.0)	0	6 (30.0)	0
Aspartate aminotransferase increased	2 (8.7)	0	6 (30.0)	0	4 (20.0)	0
Corneal epithelium defect	0	0	2 (10.0)	0	6 (30.0)	1 (5.0)

ALP, alkaline phosphatase; ALT, alanine aminotransferase; AST, aspartate aminotransferase; PPES, palmar-plantar erythrodysesthesia syndrome; TEAE, treatment-emergent adverse event; WBC, white blood cell.
